# The Effects of the Leg Position on the Nordic Hamstring Exercise Eccentric Force: A Randomized Cross-Over Study

**DOI:** 10.3390/muscles3030023

**Published:** 2024-08-18

**Authors:** Ricardo Maia Ferreira, Pedro Nunes Martins, Hugo Nunes, Luís Gonçalves Fernandes, César Ferreira Amorim, Luciano Maia Alves Ferreira

**Affiliations:** 1 Polytechnic Institute of Maia, N2i, Social Sciences, Education and Sport School, Avenida Carlos de Oliveira Campos, Castêlo da Maia, 4475-690 Maia, Portugal; pmartins@ipmaia.pt (P.N.M.); a036031@ipmaia.pt (H.N.); lfernandes@ipmaia.pt (L.G.F.); 2 Polytechnic Institute of Coimbra, Coimbra Health School, Scientific-Pedagogical Unit of Physiotherapy, Rua 5 de Outubro, São Martinho do Bispo, 3045-043 Coimbra, Portugal; 3 Sport Physical Activity and Health Research & Innovation Center (SPRINT), 4960-320 Melgaço, Portugal; 4 Master’s and Doctorate Program in Physiotherapy, University of São Paulo (Unicid), São Paulo 05508-070, Brazil; cesar.amorim@unicid.edu.br; 5 Laboratory of Physical and Functional Assessment in Physiotherapy (LAFFFi), Interdisciplinary Center of Investigation Egas Moniz (CiiEM), Quinta da Granja, 2829-511 Setubal, Portugal; lucianomaia@egasmoniz.edu.pt

**Keywords:** semitendinosus, biceps femoris, tibia, neutral, internal, external

## Abstract

Given the posterior chain configuration, it is anticipated that tibial positioning influences hamstring recruitment; medial hamstrings should be more activated during medial rotation, while lateral hamstrings should be more activated during lateral rotation. However, most studies showing this pattern have focused on concentric or isometric strength, leaving the influence on eccentric strength fairly unknown. Therefore, the aim of this study was to investigate the electromyographic response of the hamstring muscles during Nordic hamstring exercises in three leg positions: internal rotation, external rotation, and neutral. This study encompassed a randomized crossover study and used surface electromyography to analyze the activity of the biceps femoris and semitendinosus muscles during the Nordic hamstring exercise, in the three positions. Results indicated no statistically significant differences in muscle activation between positions or sides (*p* > 0.05), though small effect sizes were observed for the biceps femoris in different positions (η^2^ = 0.01–0.03). Furthermore, the internal rotation position generally elicited the highest muscle activations. Notably, biceps femoris muscles exhibited higher activations compared to semitendinosus muscles, with the greatest differences seen in the internal rotation position. This suggests that tibial rotation influences hamstring recruitment patterns; however, it was lower than expected.

## 1. Introduction

In the dynamic field of sports and exercise science, researchers are continually seeking to enhance performance and mitigate injury risks. Due to its importance in daily and sport-related activities, hamstring strength is one of the muscle properties that has received more attention in current research, both as preventive and performance-enhancing strategies [1]. It seems that hamstring strength deficit is a good predictor of hamstring injuries [2,3,4,5] and is positively related with athletic performance [3,4,5,6].

Concentric and eccentric strength exercises are frequently employed in the training regimens for these muscles [7]. To generate force, distinguished mechanisms are involved, where eccentric actions occur due to the active lengthening of the fascicles, and concentric actions due to their active shortening [8]. Although both types are commonly used, studies have demonstrated greater muscular gains through eccentric over concentric training [9,10,11].

One of the most popular eccentric hamstring exercises among athletes and coaching/medical staff is the Nordic hamstring exercise (NHE) [7]. The NHE, also referred to as the Russian hamstring exercise [12], was first reported in research in 2001 by the Brockett et al. [13] study, but its popularity surged notably after the Mjølsnes et al. [14] study. The NHE usually involves two individuals, where one person performs the exercise by resisting a forward-falling motion from a kneeling position, using their hamstring muscles to maximize loading during the eccentric phase; the second person stabilizes the lower legs/feet with their hands, ensuring maximal hamstring recruitment [15].

The hamstrings are a bi-articular muscular group, localized in the posterior thigh, and comprise three separate muscles, situated in either the medial (semitendinosus [ST] and semimembranosus [SM]) or lateral (biceps femoris [BF]) compartment [16,17]. All three muscles originate from the ischial tuberosity of the pelvis, but they have different insertion sites; the ST and SM insert on the medial surface of the tibia, while the BF inserts on the lateral supracondylar line of the tibia and fibular head [18]. They are responsible for knee flexion and hip extension on the sagittal plane, as well as the hip and knee (tibial) joint rotation on the transverse plane, with medial hamstrings facilitating internal rotation and the lateral hamstrings facilitating external rotation [16,19,20,21,22]. Despite their importance in both joints, the hamstrings are anticipated to have a greater influence on the knee compared to the hip. For example, the Park and Lim [21] study found that the EMG activity of the medial and lateral hamstrings ranged from 56.8% to 62.5% during maximal isometric knee flexion (as knee flexors), compared to 18.3% to 36.0% during maximal isometric hip extension (as hip extensors).

Given the posterior chain configuration, it is anticipated that tibial positioning influences the recruitment pattern of the hamstring muscles. Specifically, the medial hamstrings are expected to be more activated during internal rotation, while the lateral hamstrings are expected to be more activated during external rotation [21,23,24,25,26]. However, most studies investigating this feature have focused on isometric strength [23,24,25,27,28], or included a post-surgery sample [22], leaving the influence on eccentric strength fairly unknown. Moreover, previous research has emphasized the significance of hip, knee, and ankle angles in the NHE, due to their potential influence on the outcomes [29,30,31,32,33,34]. Additionally, despite existing guidelines for knee placing for this exercise [35,36], information regarding tibial positioning is still limited. Therefore, the aim of this study is to investigate the electromyographic response of the hamstring muscles during the NHE in three leg positions (internal rotation, external rotation, and neutral). Although information on the influence of tibial positioning on hamstring muscle activation during eccentric strength exercises is scarce, this study hypothesizes that during the NHE, the ST and SM muscles will be more activated with internal tibial rotation, while the BF will be more activated with external tibial rotation.

## 2. Material and Methods

### 2.1. Sample

The study included 13 athletes from the national under-20 women’s volleyball team. Participants were selected based on their active participation in regular team training sessions. The eligibility criteria for participation included the following: an RPE below 5 (0–10); practicing federated volleyball; not having acute (less than 6 months) or chronic injuries in the lower limbs, or surgeries; having their sports medical exam active and updated; not taking regular medication; not having diabetes or menstruating; not having hormonal, hepatic, renal, gastrointestinal, cardiovascular, respiratory, or neuronal health disorders; not have consumed caffeine, alcohol, or drugs in the last 72 h; and being familiarized with the NHE. Furthermore, for the study participation, all participants provided informed consent before participating in the study.

### 2.2. Study Design

This investigation employed a randomized cross-over design [37] to assess electromyographic activity during the NHE in three different lower leg positions. The study was conducted at the institutional laboratory for data collection, with environment controls (namely, surface, noise, electronic devices, temperature, and humidity). The study was conducted according to the Declaration of Helsinki guidelines, and the ethical approval was obtained from the Polytechnique Institute of Coimbra Review Board.

### 2.3. Electromyography (EMG) Procedures

**Figure 1:**
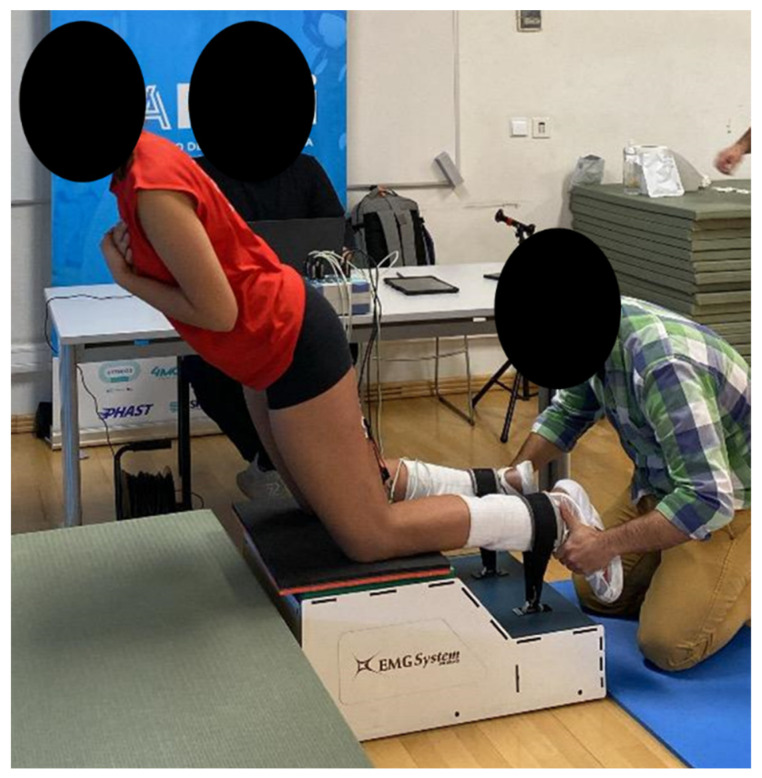
EMG device and test execution.

EMG signals were recorded using a 4-channel biological signal acquisition system model SAS2000V4-WiFi with EMGNordic—Integrated Kinetic Platform (EMG System, Brazil). Circular electrodes (Ag/AgCl—Medical Trace), 10 mm in diameter, were used to record surface EMG signals from the BF and ST muscles (Figure 1). Electrode localization and orientation adhered to the guidelines outlined in the Surface ElectroMyoGraphy for the Non-Invasive Assessment of Muscles [38]. The skin was shaved, abraded, and cleaned with 70% ethyl alcohol to reduce impedance. Participants were instructed to perform the NHE with standardized form and tempo. Three valid repetitions were recorded in each position. A 10 s inter-repetition and a 5 min rest period between evaluated positions were given, in order to counteract the accumulation of excessive muscular fatigue across sets and to ensure the preservation of both exercise quality and intensity.

To ensure that the exercise was performed with the correct technique, the movement was videotaped, and the alignment analyzed in real time using specialized software. If an impaired technique was detected (e.g., inadequate hip flexion, lumbar lordosis, movement speed), the participant was requested to repeat the exercise, until reaching three valid repetitions in each position. Before collecting the data, a repetition pre-test was allowed, to familiarize the participant with the procedures and to make any necessary corrections in the exercise execution technique. Subsequently, the data were processed using specific software for acquisition and analysis (Toolbox BR V1.0, EMG System, Brazil), along with a converting plate A/D 16 bit signal, converting analog to digital signals with a sampling frequency of anti-aliasing 2.0 kHz for each channel. Signal processing involved full-wave rectification, linear coverage via a fourth-order Butterworth filter (cutoff frequency of 5 Hz), and normalization in both time and amplitude. The amplitude was normalized from the mean amplitude of the full-wave rectified EMG signal [39]. The root mean square (RMS) was calculated for each muscle during the specific NHE positions to determine the amplitude of the signal.

### 2.4. Nordic Exercises

**Figure 2:**
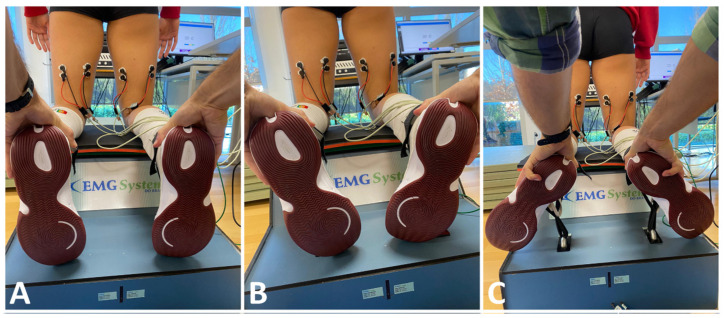
Different leg positions tests. (**A**)—Neutral; (**B**)—Maximum internal rotation; (**C**)—Maximum external rotation.

Participants were evaluated in the NHE in three different lower leg positions: neutral, maximal internal rotation, and maximal external rotation (Figure 2). These exercises were conducted on the EMG device (25 cm above ground), allowing for controlled and standardized execution. The exercise execution followed established guidelines, with participants positioned accordingly [35,36]. The participants start in a kneeling position, with the torso from the knees upward held rigid and straight. Furthermore, the knees were aligned with the pelvis, trunk, shoulder, neck, and head, with shoulder-width spacing. A rigid belt was positioned in the intermalleolar area to stabilize the lower limb and prevent unnecessary movements. The feet had no interaction with the device (contact free), remaining in a neutral position in all tests. It was suggested for participants to keep their hands facing forward and their elbows pointing down, ready to buffer the fall, during all the test’s executions.

Additionally, cushions were strategically placed in the falling area for safety reasons. For the eccentric phase of the exercises, participants were instructed to perform a controlled downward movement of the trunk as slowly as possible (approximately, 10 s—10°/s) in the direction to the ground, controlling the speed with the hamstring’s strength, and maintaining a straight neutral spine, avoiding hip drop and keeping the arms still. This action was performed until the participant could no longer withstand the torque around their knees caused by the increasing moment arm of their weight as they leaned forwards. The NHE during execution was supervised, and feedback was given to the participants about their speed and posture. For the three variations, one of the researchers held or rotated the leg, ensuring that it remained in the test position throughout the exercise.

The order of the positions was randomized to mitigate any potential order effects. The randomization was performed in the Random online software (random.org, accessed on 12 July 2024), and the participants were blind to the corresponding group. Prior to the trials, each participant underwent a standardized warm-up, including 5 min of low-intensity running, and a series of bodyweight dynamic movements such as squats, walking lunges, hip bridges, single leg bridges with knee extension–flexion (2 sets of 15 repetitions, for each exercise). A warm-up of 2 sets of 3 submaximal bilateral NHEs was performed prior to the trials per person.

### 2.5. Statistical Analysis

For the statistical analysis, the SPSS 28 program (IBM, New York, NY, USA) was used. Descriptive statistics were calculated and reported as mean ± standard deviation. The Kolmogorov–Smirnov test, Q-Q, and graphical analysis of the skewness and kurtosis were used for the testing of normality, with the Levene’s test for equality of variances. The ANOVA test was conducted to assess any significant differences between the positions, with a Tukey post-hoc test. Effect sizes were determined with the eta square and interpreted as small (0.01 ≤ η^2^ < 0.06), medium (0.06 ≤ η^2^ < 0.14), and large (η^2^ ≥ 0.14). Additionally, between-muscle statistical comparisons and their effects sizes were calculated (Cohen’s d—0.0 ≤ d < 0.2 [very small]; 0.2 ≤ d < 0.5 [small]; 0.5 ≤ d < 0.8 [medium]; d ≥ 0.8 [large]) [40].

## 3. Results

**Figure 3:**
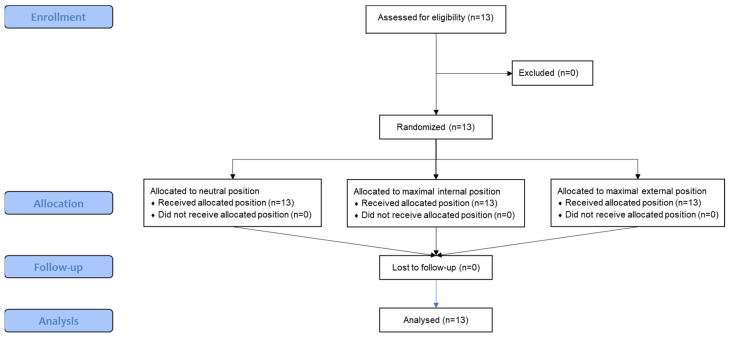
CONSORT flow diagram showing the study’s recruitment and randomization.

Out of the initially recruited 13 athletes, none were excluded based on eligibility criteria and none were lost in the allocation, follow-up, or analysis phases (Figure 3).

Accordingly, the sample exhibited a mean age of 17.3 ± 0.6 years, paired with an average volleyball experience of 7.3 ± 3 years. The team’s weight averaged at 68.5 ± 7.2 kg, while their mean height measured 1.79 ± 0.06 m, resulting in an average BMI of 21.4 ± 1.7 kg/m^2^. The right lower limb emerged as the predominant dominant side, with 11 athletes (84.6%) favoring it. Additionally, at the time of data collection, players displayed a low level of fatigue, as indicated by a mean RPE of 2.8 ± 1.2. For more detailed information, see Table 1.

Overall, the results indicated no statistically significant differences (*p* > 0.05) between positions, hamstring muscles, or sides.

Nevertheless, small effect sizes (η^2^ = 0.01 to 0.03) were found between positions in BF left, BF Right, ST Right, BF (Right + Left), and Left side (BF + ST). No effects were found for ST Left, ST (Right + Left), or Right side (BF + ST). The Internal position generally showed the highest activations (227.84 to 203.38), except for BF Right (Neutral—219.14 ± 57.49). The largest mean differences were most frequently between the Internal and Neutral positions (BF Right—25.86; Left-side—14.93; ST Right—13.26; ST (Left + Right)—8.64; ST Left—4.01), followed by differences between the Internal and External positions (BF (Left + Right)—14.69; Right-side—7.65), and between the Neutral and External positions (BF Right—12.53).

When comparing the left-side hamstring muscles (BF vs. ST), very small effect sizes were found in the Neutral and External positions (d = 0.07 and d = 0.04, respectively), with a large effect size in the Internal position (d = 0.84). For the right-side hamstring muscles, very small (d = 0.02), small (d = 0.24), and medium effect sizes (d = 0.49) were found in the External, Neutral, and Internal position, respectively. The BF muscles consistently exhibited higher EMG activations in comparison to the ST muscles, showing a very small effect size in the External position (d = 0.01), a small effect size in the Neutral position (d = 0.20), and a medium effect size in the Internal position (d = 0.66). A similar pattern emerged when comparing the Right- to the Left-side, where very small effects were found in the External position (d = 0.12), small effects in the Neutral position (d = 0.28), and medium effects in the Internal position (d = 0.56).

**Figure 4:**
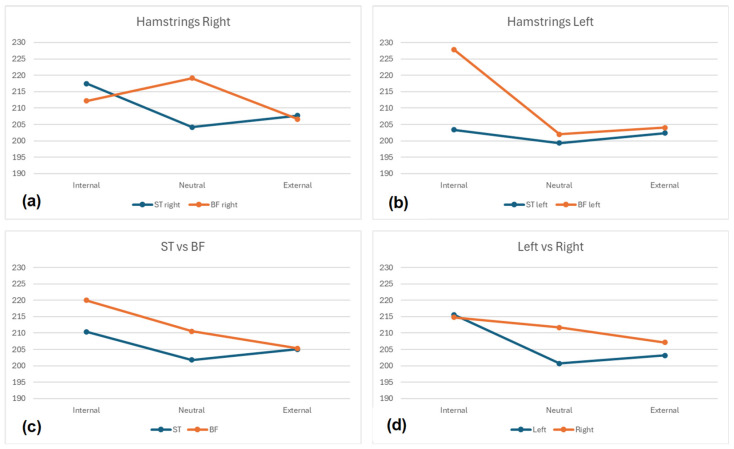
Mean EMG Activity. (**a**) Neutral, Internal, and External activations for right side Semitendinosus and Biceps Femoris muscles; (**b**) Neutral, Internal, and External activations for left side Semitendinosus and Biceps Femoris muscles; (**c**) Neutral, Internal, and External activations for the left and right Semitendinosus muscles, and the left and right Biceps Femoris muscles; (**d**) Neutral, Internal, and External activations for the left and right hamstrings.

**Figure 5:**
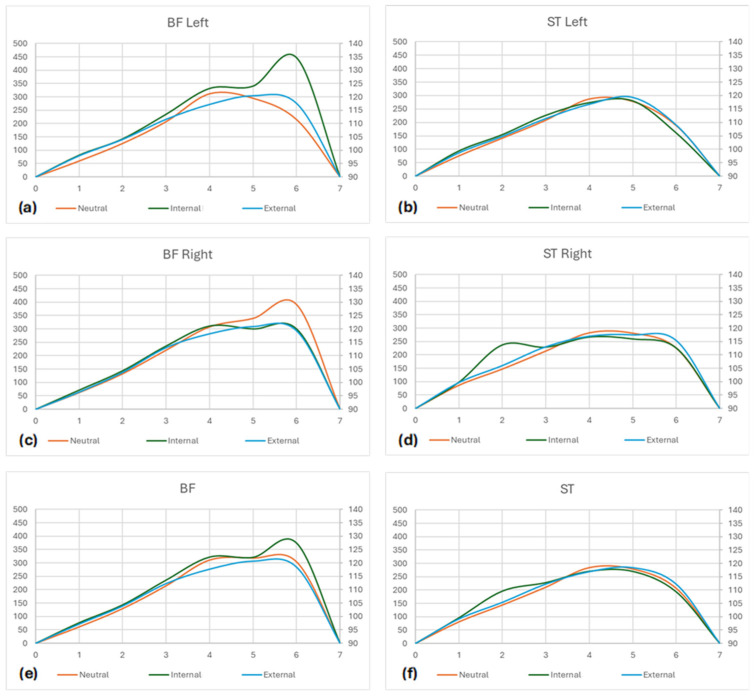

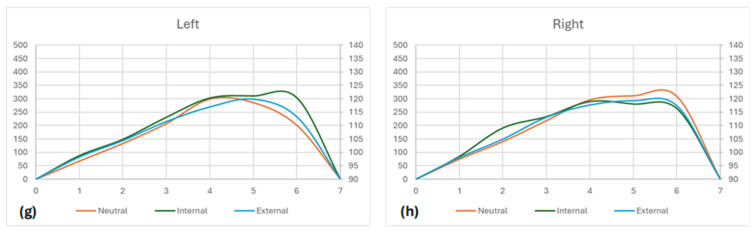
Activity curves (EMG × Time × Range of Motion). (**a**) Neutral, Internal, and External curves for the Biceps Femoris muscle of the left thigh; (**b**) Neutral, Internal, and External curves for the Semitendinosus muscle of the left thigh; (**c**) Neutral, Internal, and External curves for the Biceps Femoris muscle of the right thigh; (**d**) Neutral, Internal, and External curves for the Semitendinosus muscle of the right thigh; (**e**) Neutral, Internal, and External curves for the Biceps Femoris muscles of both sides; (**f**) Neutral, Internal, and External curves for the Semitendinosus muscles of both sides; (**g**) Neutral, Internal, and External curves for the Biceps Femoris and Semitendinosus muscles of the left thigh; (**h**) Neutral, Internal, and External curves for the Biceps Femoris and Semitendinosus muscles of the right thigh.

More detailed information is provided in Table 2 and Figure 4 and Figure 5.

## 4. Discussion

The present study aimed to investigate the electromyographic response of the hamstring muscles during the NHE in three different lower leg positions: neutral, internal rotation, and external rotation. It was anticipated that during tibial external rotation, the BF would be in a more lengthened position, potentially enhancing its activation compared to the neutral or internally rotated positions. Similarly, it was expected that the ST muscle would exhibit increased activation during internal rotation. For example, Mohamed et al. [23] found that during medial rotation the ST and SM muscles exhibited higher mean activity (109% and 107%, respectively) compared to lateral rotation (95% and 89%, respectively), while the long and short head of the BF muscles demonstrated greater activity during lateral rotation (110% and 108%, respectively) versus medial rotation (93% and 97%, respectively). Additionally, Fiebert, et al. [25] found 40% more activity in the medial hamstring with medial tibial rotation, while the lateral hamstrings had a 59% increase with lateral tibial rotation. Also, Beyer et al. [24] found that the lateral hamstrings had a higher amount of activation when the tibia was externally rotated or in the neutral position as compared to internally rotated and vice versa (*p* < 0.001). However, in the present study, it was found that the NHE performed in the internal rotation elicited significantly greater activation of the overall hamstring muscles (especially BF) compared to the neutral and external rotation positions. Nevertheless, no statistically significant differences (*p* > 0.05) between positions, hamstring muscles, or sides were found. Similar results have been reported in other studies [28]. These differences found between the studies can be attributed to factors related to exercise execution and personal/sports biomechanical patterns.

The NHE is an eccentric exercise where the hamstrings are progressively stretched while simultaneously contracting to slow down the downward movement. This contraction type can influence the hamstrings’ contraction patterns differently compared to concentric or isometric contractions. For example, as previous discussed, Lynn and Costigan [26] also found that active external rotation of the foot selectively activates the lateral hamstrings and active internal rotation of the foot selectively activates the medial hamstrings in hamstring exercises such as the hamstring curl, hamstring bridge, and one-legged deadlift. However, during the eccentric phase of the one-legged deadlift, the medial–lateral hamstring activation ratio during internal rotation was not significantly different from that during the normal or externally rotated positions. One proposed reason is that the movement might stimulate the Golgi Tendon Organ to produce some aberrant muscular activity in the subjects (especially those with tight hamstrings), as their function is to protect the muscle and its connective tissue from injury by inducing reflex inhibition of the alpha motor neuron when excessive tension is detected in the muscle or tendon.

Another explanation related to exercise execution is that during the NHE, tibial rotation and stabilization are passively restrained by the evaluator, whereas in other exercises the tibia is either unrestricted or must actively maintain its position. For example, in the Beuchat and Maffiuletti [22] study, among the three exercises studied, only the prone leg curl exercise showed a significant increase in EMG activity of the medial hamstrings with internal foot rotation (concentric: +8.7% MVC; eccentric: +5.9% MVC; *p* < 0.01; d = 0.88–0.99) and a decrease with external rotation (concentric: 5.8% MVC; eccentric: 5.2% MVC; *p* < 0.05; d = 0.67–0.92). In the other exercises, namely the NHE and single-leg bridge, the positions did not significantly change the hamstring EMG activity. In the prone leg curl exercise, the foot rotations have to be performed actively, while in the NHE, the foot is passively restrained by the evaluator. Consequently, during the NHE, the hamstring muscles are primarily activated as knee flexors and not, or minimally, as knee rotators. This can further explain as in the NHE the subject has to perform the exercise in a kneeling position, the anterior part of the knee is under compression, potentially modifying patellar tendon activity. As previous shown in the literature [20], the patellar tendon is the strongest rotator of the tibia, followed by the hamstrings. Therefore, with the reduced patellar tendon activity due to the exercise position, the hamstrings may be less affected by the tibia rotation.

In the present study, overall small effect sizes were found in the BF muscles across the three lower leg positions, while the ST muscles showed similar activation levels. This pattern is consistent with findings from other studies. For instance, Jónasson et al. [27] found that when isometric knee flexion was coupled with lateral rotation, activation levels were similar for the medial and lateral hamstrings (1.01 ± 0.31 ratio). In contrast, with medial rotation, a significant drop in lateral hamstring activation led to dissimilar activation levels between the two muscle groups (1.64 ± 0.88 ratio). On average, knee flexor strength measures were 9.1% greater when flexion was coupled with tibial lateral rotation compared to medial rotation, reflecting significantly greater activation levels of the lateral hamstrings while the medial hamstrings were largely unaffected by tibial rotation. Therefore, it appears that the hamstrings have a lower impact on the tibia rotation than anticipated. However, being bi-articular muscles, the hip position may also influence their EMG activation patterns. Park and Lim [21], as expected, found that during the maximal isometric knee flexion, EMG activity of medial hamstrings was 60.9 ± 16.7% MVIC with tibial internal rotation and 56.8 ± 16.0% MVIC with tibial external rotation (*p* = 0.211), while for the lateral hamstrings it was 58.7 ± 15.1% MVIC with tibial internal rotation and 62.5 ± 15.1% MVIC with tibial external rotation (*p* = 0.262). Therefore, although some differences were found, they were not statistically significant. However, during maximal isometric hip extension, EMG activity of the medial hamstrings was 24.0 ± 13.4% MVIC with hip internal rotation and 18.3 ± 11.0% MVIC with hip external rotation (*p* = 0.001), while for the lateral hamstrings it was 25.5 ± 11.6% MVIC with hip internal rotation and 36.0 ± 21.2% MVIC with hip external rotation (*p* = 0.001). This suggests that the hamstrings are more influenced by hip rotation than knee rotation. Given that volleyball athletes often have a toe-out gait pattern due to the demands of the sport, it is expected that the lateral hamstrings are more consistently activated compared to the medial hamstrings [41,42], explaining why, in the present study, the BF almost always showed higher EMG values than the ST.

## 5. Limitations

It is worth noting that the current study focused on the acute electromyographic response during the Nordic exercise in a specific sports sample. Other samples may have a different activation pattern. Furthermore, in the study only female volleyball players were accessed. Although no differences between sexes were expected to be found in the hamstring muscle architecture [43], a male population should also be explored. Furthermore, an increased sample size is also advised, as in the current study, it may limit the analysis. Finally, since tibia positioning was performed manually, some errors may have occurred. Future studies should consider using mechanical methods to improve accuracy.

## 6. Conclusions

In conclusion, the findings of this study suggest that the NHE exercise performed in a maximal internal rotation position elicits greater activation of the BF and overall hamstring musculature compared to the neutral and maximal external rotation positions. The ST muscles are less affected by the tibia position, as showed similar activation levels.

## Figures and Tables

**Table 1:** Sample characterization (n = 13).

ID	Age (Years)	Weight (kg)	Height (m)	BMI (kg/m^2^)	RPE (0–10)	Volleyball Years	Dominate Side	Volleyball Position
1	18	75.1	1.86	21.7	2	10	Right	Outside Hitter
2	16	62.8	1.71	21.5	4	4	Right	Libero
3	17	73.2	1.85	21.4	3	9	Right	Opposite Hitter
4	18	69.6	1.82	21.0	2	7	Right	Setter
5	17	74.8	1.85	21.9	4	7	Left	Middle Blocker
6	18	57.9	1.76	18.7	1	14	Right	Outside Hitter
7	17	68.9	1.86	19.9	4	2	Right	Middle Blocker
8	17	57.8	1.68	20.5	2	9	Right	Libero
9	17	57.4	1.76	18.5	4	6	Left	Setter
10	17	68.3	1.70	23.6	3	5	Right	Middle Blocker
11	17	78.2	1.84	23.1	1	6	Right	Opposite Hitter
12	18	73.7	1.76	23.8	2	9	Right	Setter
13	18	72.5	1.81	22.1	4	7	Right	Setter
Mean ± SD; n (%)	17.3 ± 0.6	68.5 ± 7.2	1.79 ± 0.06	21.4 ± 1.7	2.8 ± 1.2	7.3 ± 3	Right—11 (84.6%) Left—2 (15.4%)	Setter—4 (30.8%) Opposite Hitter—2 (15.4%) Outside Hitter—2 (15.4%) Middle Blocker—3 (23.1%) Libero—2 (15.4%)

Abbreviation: BMI, body mass index; ID, identification; kg, kilograms; m, meters; n, frequency; RPE, rate of perceived exertion; SD, standard deviation.

**Table 2:** Comparison of EMG across different positions and muscles.

	Positions			Statistics	Effect Size
	Neutral	Internal	External	*p*	η^2^
BF Left	201.98 ± 64.69	227.84 ± 78.09	204.02 ± 63.10	0.57	0.03
ST Left	199.37 ± 71.59	203.38 ± 71.92	202.35 ± 79.85	0.99	0.00
*p*	0.82	0.32	0.88		
d	0.07	0.84	0.04		
BF Right	219.14 ± 57.49	212.16 ± 61.25	206.61 ± 67.57	0.88	0.01
ST Right	204.19 ± 59.26	217.45 ± 100.74	207.69 ± 68.03	0.90	0.01
*p*	0.41	0.85	0.95		
d	0.24	0.49	0.02		
BF (Left + Right)	210.56 ± 53.62	220.00 ± 62.89	205.31 ± 63.11	0.82	0.01
ST (Left + Right)	201.78 ± 63.11	210.41 ± 83.08	205.02 ± 69.77	0.95	0.00
*p*	0.74	0.68	0.98		
d	0.20	0.66	0.01		
Left (BF + ST)	200.68 ± 65.20	215.61 ± 62.00	203.18 ± 69.17	0.83	0.01
Right (BF + ST)	211.67 ± 49.29	214.81 ± 67.15	207.15 ± 61.16	0.95	0.00
*p*	0.33	0.94	0.67		
d	0.28	0.56	0.12		

## Data Availability

Data can be assessed by emailing the corresponding author.
